# Whole Body Vibration Improves Cognition in Healthy Young Adults

**DOI:** 10.1371/journal.pone.0100506

**Published:** 2014-06-20

**Authors:** G. Ruben H. Regterschot, Marieke J. G. Van Heuvelen, Edzard B. Zeinstra, Anselm B. M. Fuermaier, Lara Tucha, Janneke Koerts, Oliver Tucha, Eddy A. Van Der Zee

**Affiliations:** 1 University of Groningen, University Medical Center Groningen, Center for Human Movement Sciences, Groningen, The Netherlands; 2 Department of Clinical and Developmental Neuropsychology, University of Groningen, Groningen, The Netherlands; 3 Center of Behaviour and Neuroscience, Department of Molecular Neurobiology, University of Groningen, Groningen, The Netherlands; University of Sao Paulo, Brazil

## Abstract

This study investigated the acute effects of passive whole body vibration (WBV) on executive functions in healthy young adults. Participants (112 females, 21 males; age: 20.5±2.2 years) underwent six passive WBV sessions (frequency 30 Hz, amplitude approximately 0.5 mm) and six non-vibration control sessions of two minutes each while sitting on a chair mounted on a vibrating platform. A passive WBV session was alternated with a control session. Directly after each session, performance on the Stroop Color-Block Test (CBT), Stroop Color-Word Interference Test (CWIT), Stroop Difference Score (SDS) and Digit Span Backward task (DSBT) was measured. In half of the passive WBV and control sessions the test order was CBT-CWIT-DSBT, and DSBT-CBT-CWIT in the other half. Passive WBV improved CWIT (p = 0.009; effect size r = 0.20) and SDS (p = 0.034; r = 0.16) performance, but only when the CBT and CWIT preceded the DSBT. CBT and DSBT performance did not change. This study shows that two minutes passive WBV has positive acute effects on attention and inhibition in young adults, notwithstanding their high cognitive functioning which could have hampered improvement. This finding indicates the potential of passive WBV as a cognition-enhancing therapy worth further evaluation, especially in persons unable to perform active forms of exercise.

## Introduction

There is growing evidence that physical exercise has positive effects on cognition [1]. Especially executive functions benefit from physical exercise [2]. Executive functions are a set of cognitive processes that regulate, manage and control other cognitive processes in order to achieve a goal, such as planning, working memory, mental flexibility, inhibition, attention, problem-solving and multi-tasking [3]. Executive functions are associated with the prefrontal cortex [4]. People who are not able to perform physical exercise, however, cannot fully benefit from the positive effects of physical exercise on executive functions. Therefore, whole body vibration (WBV) may be an alternative. WBV is the undergoing of mechanical vibrations produced by a vibrating platform to improve physical fitness. Often exercises are performed while standing on the vibrating platform. Besides this active form of WBV, it is also possible to undergo WBV without performing exercises. Such a passive form of WBV, referred to as passive WBV hereafter, may be a suitable physical therapy for improving executive functions in persons who are not able to perform physical exercise. However, the effects of passive WBV on executive functions have not been investigated yet. Therefore, this study investigated the acute effects of passive WBV on executive functions in healthy young adults.

It has been shown that active WBV has positive acute effects on human physiology. For example, oxygen uptake, heart rate, diastolic blood pressure and muscle activity are increased during active WBV compared to values during the performance of the same exercises without WBV [5]–[7]. The working mechanism behind the acute physiological effects of WBV, however, is not clear yet [6]. It is hypothesized that WBV stimulates muscle spindles, leading to a reflex response of the muscle [8]. This may enhance muscle activity, resulting in increased oxygen uptake and heart rate (both reflecting increased energy expenditure).

Besides possible excitation of muscle spindles, WBV stimulates vibration-sensitive mechanoreceptors in the skin, such as the Meissner corpuscles sensitive to 10–80 Hz vibrations and specifically to 30–40 Hz vibrations [9]–[11]. Afferent signals of cutaneous mechanoreceptors are transmitted to the primary somatic sensory cortex [12]. The sensory association areas have a direct and indirect connection to the prefrontal cortex [13], a region strongly involved in cognitive processing [4], [14]. The indirect pathway involves the limbic system (e.g. the amygdala and the hippocampus, regions important in learning and memory), which can mediate the influence of the sensory association areas on the prefrontal cortex [13]. Furthermore, the amygdala also has projections to non-thalamic nuclei (e.g. the cholinergic nuclei of the basal forebrain) that have diffuse connections to a number of brain regions [13]. Thus, sensory stimulation may influence neurotransmission in sensory brain regions as well as the prefrontal cortex, hippocampus, amygdala and other brain regions. Therefore, a passive WBV session may have positive acute effects on cognition by sensory stimulation.

Empirical support for positive effects of WBV on cognition is provided by chronic WBV studies in animals. In these studies mice were placed in a cage connected to a vibrating platform and exposed to 10 minutes WBV (30 Hz–1.9 g) per day for five weeks. Results showed that daily exposure to WBV for five weeks significantly improved cognition (e.g. spatial memory) in young and aged mice compared to non-vibrated control mice [15], [16]. Furthermore, the expression of the immediate early gene c-fos was enhanced in brain areas involved in sensorimotor and learning and memory functions (while leaving other brain regions largely unchanged) of mice exposed to WBV [17]. C-fos is up-regulated by an increase in neurotransmission and activates genes involved in the production of proteins necessary for neuronal plasticity and long-term memory [18], [19]. The brain regions in which c-fos expression was enhanced are the same brain regions in which sensory stimulation is assumed to increase neurotransmission (sensory brain regions, prefrontal cortex, hippocampus). In addition, we recently demonstrated that WBV significantly increased the activity of the mouse forebrain cholinergic system (unpublished data). These findings suggest that sensory stimulation induced by WBV is an essential part of the mechanism underlying the improved cognitive performance in mice.

The neuroanatomical connections between mechanoreceptors and cognition-related brain regions (e.g. the prefrontal cortex), as well as the positive effects of WBV on cognition and neurotransmitter systems in mice, suggest that passive WBV may positively influence neurotransmission in a wide range of human brain regions. However, since the sensory brain regions have a direct and indirect connection to the prefrontal cortex, we expected that sensory stimulation by passive WBV would in particular affect executive functions. Therefore, the general aim of this study was to investigate the acute effects of passive WBV on executive functions in healthy young adults. Because sensory stimulation by passive WBV may positively influence neurotransmission in the prefrontal cortex, we hypothesized that passive WBV would improve executive functions.

## Methods and Results

### Ethics Statement

The Institutional Review Board (the Ethical Committee Psychology) of the Psychology Department of the University of Groningen approved the study. The study protocol conforms to the Helsinki declaration. All participants signed an informed consent before the experiment started.

### 2.1 Study Design

First a pilot study was performed to investigate the acute effects of passive WBV on executive functions in a small sample of young adults. An additional aim of the pilot study was to identify the optimal WBV frequency and amplitude for acute cognitive enhancement. After the pilot study, the main study was performed. The aim of the main study was to investigate the acute effects of passive WBV on executive functions in a large sample of young adults by using the optimal frequency and amplitude for acute cognitive enhancement as determined by the pilot study.

### 2.2 The Pilot Study

#### 2.2.1 Participants

In the pilot study 12 healthy young adults (4 females and 8 males; age: 22.8±1.5 years; mass: 68.3±9.3 kg; height: 1.81±0.1 m; amount of exercise: 5.6±3.0 hours/week) voluntarily participated without receiving a reward for participation. The participants were students from the University of Groningen, the Netherlands. Participants did not have red-green color deficiency or any history of neurological or psychiatric disease.

#### 2.2.2 Whole body vibration chair

**Figure 1:**
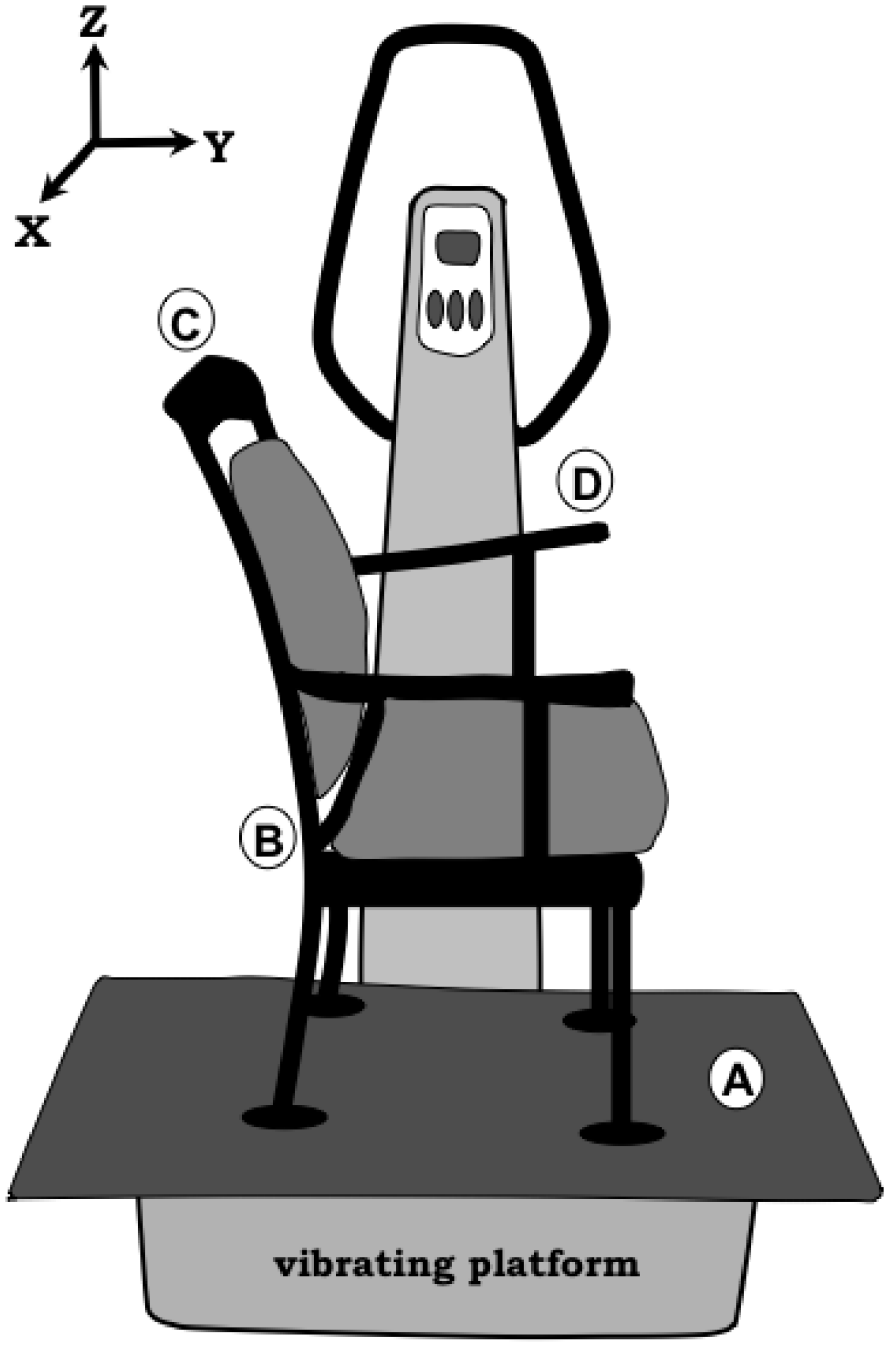
The WBV chair. Accelerations were measured at location A – D to determine the actual vibration frequency and amplitude (see Table 1).

A commercially available vibration device (Vibe 300 from Tonic Vibe, Nantes, France) was used in this study. A wooden plate (0.5 m×0.9 m×0.02 m) was mounted on the vibrating platform to enlarge the platform of the device (Figure 1). In order to apply passive WBV, a chair (with armrests and a seating area of soft material) was firmly mounted on the wooden plate to control activity and movement of participants (Figure 1). The vibration platform generated mainly vertical sinusoidal vibrations. According to the manufacturer, the vibration frequency could be adjusted with 1 Hz increments from 15 Hz to 60 Hz and the vertical vibration amplitude could be set at 2 mm or 4 mm. In this study accelerometers (tri-axial accelerometers, model 3093B, Dytran Instruments Inc, Chatsworth, CA, USA) were used to determine the actual frequency and amplitude of the vibration platform. With an average spectrum analysis of the acquired acceleration signal (using OR36 Signal Analyzer and NVGATE software version 7.00 from OROS SA, Grenoble, France) the actual frequency (Hz) and amplitude (mm) of the vibration platform were determined. We found that the actual frequency was equal or very similar to the frequency set on the vibration device (Table 1). However, the actual amplitude was lower than the amplitude reported by the manufacturer (Table 1).

**Table 1:** The actual vibration frequency and amplitude of vertical displacement for different locations (A–D in Figure 1) and different frequency and amplitude settings on the vibration device.

WBV condition	Settings WBV device (Hz, mm)	Actual frequency and amplitude (Hz, mm)			
		*Location A*	*Location B*	*Location C*	*Location D*
1	20, 2	20, 0.38	20, 0.45	20, 0.38	20, 0.50
2	30, 2	30, 0.10	30, 0.19	30, 0.08	30, 0.62
3	40, 2	38, 0.16	38, 0.29	38, 0.26	38, 0.28
4	50, 2	48, 0.28	48, 0.33	48, 0.33	48, 0.33
5	60, 2	58, 0.38	58, 0.36	58, 0.45	58, 0.19
6	20, 4	20, 0.71	20, 0.72	20, 0.76	20, 0.74
7	30, 4	30, 0.44	30, 0.44	30, 0.64	30, 0.50
8	40, 4	38, 0.39	38, 0.46	38, 0.42	38, 0.47
9	50, 4	48, 0.46	48, 0.51	48, 0.53	48, 0.58
10	60, 4	58, 0.59	58, 0.60	58, 0.81	58, 0.33

The actual frequency and amplitude were calculated based upon acceleration data. Accelerations were measured without a person on the WBV chair.

#### 2.2.3 Study design and procedures

**Figure 2:**
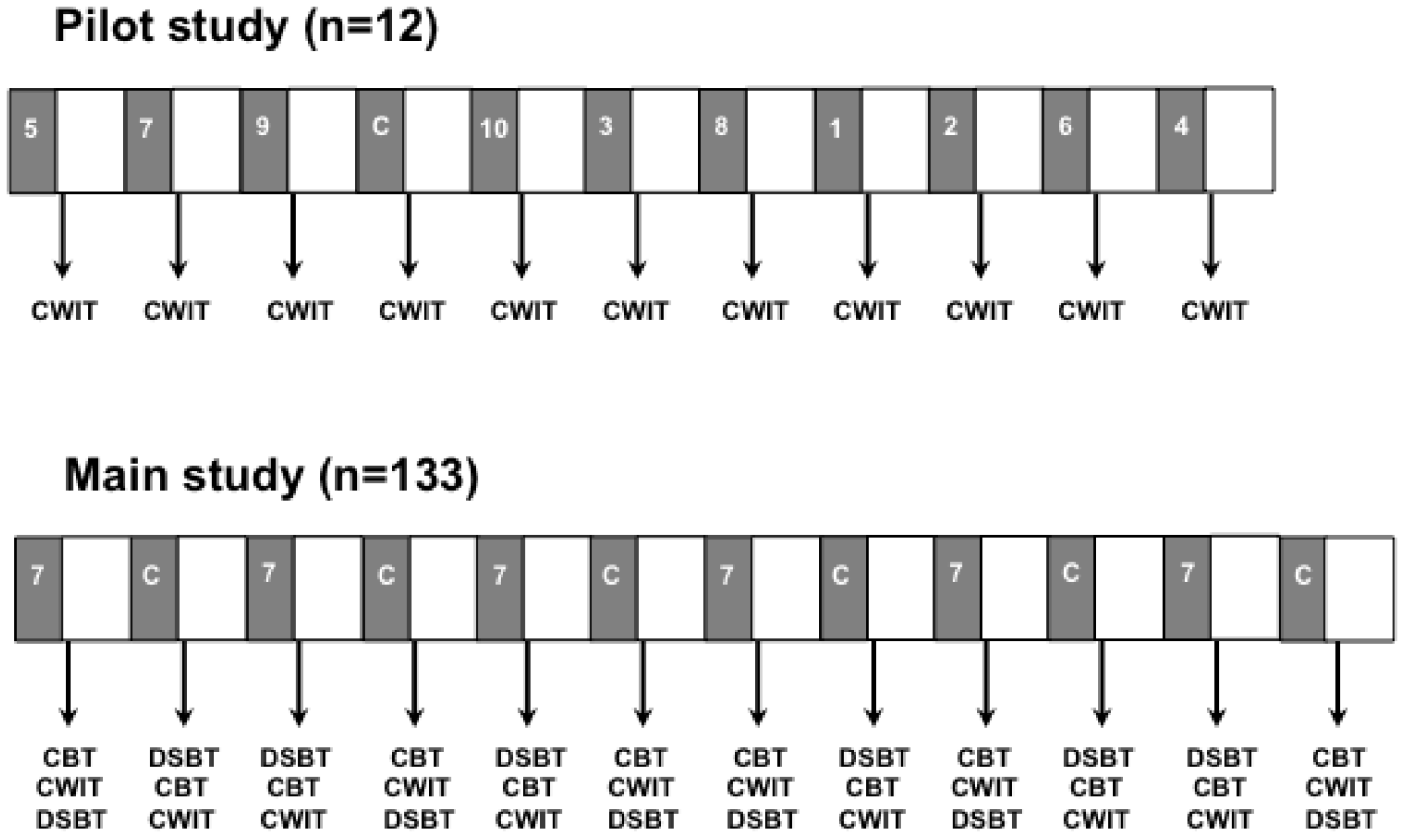
An example of a session order used in the pilot study and the main study. Grey blocks represent the WBV conditions (WBV conditions 1–10 in pilot study and WBV condition 7 in main study; see Table 1 for frequency and amplitude per WBV condition) or control condition (C). White blocks represent the rest periods. Executive functions were assessed immediately after each WBV and control session by using the Color-Word Interference Test (CWIT) in the pilot study, and the CWIT, Color-Block Test (CBT) and Digit Span Backward task (DSBT) in the main study.

Participants participated in 10 different passive WBV conditions (see Table 1 for the vibration frequency and amplitude per WBV condition) and a control condition while sitting on the WBV chair. In the control condition no vibration was applied. Three random orders of the 11 conditions were generated. By reversing these three orders three additional orders were created to counterbalance the design to avoid order effects. To obtain a total of 12 orders (one order for each participant), each of the six orders was applied twice. The 12 orders of the 11 conditions were randomly allocated to the 12 participants. Figure 2 shows an example of a condition order used in the pilot study. Each condition (passive WBV or control) lasted two minutes, immediately followed by assessment of executive functions. After the cognitive assessment participants had three minutes rest while sitting on the WBV chair (without vibration) before the next condition started.

Participants were instructed to sit in an upright position against the back of the chair throughout the whole experiment, with their arms on the armrests and their feet on the wooden plate. In addition, they were instructed to keep body movements to a minimum.

#### 2.2.4 Measurements

In the pilot study executive functions (attention and inhibition) were assessed with the Color-Word Interference Test (CWIT) of the Stroop Color-Word Interference task [4], [20]. In the CWIT a card with 20 color names (blue, red, orange, green or black printed in Dutch) was presented to the participant. Each word was printed in one of five possible colors (blue, red, orange, green or black). However, the ink color of each word was different from the color name (e.g. the word blue was printed in red). Participants were asked to name the ink color of the 20 words as fast as possible, thereby ignoring the written color name. The time (in seconds) needed to accomplish the test was measured. The participants practiced the CWIT once before the experiment started. Twelve parallel versions (one version for practice and 11 versions for assessments during the experiment) of the same CWIT (20 color names, five colors) were designed. Three random orders were generated of the 12 CWIT versions. Three additional orders were created by reversing the three random orders to counterbalance the order of the 12 CWIT versions across participants. Each of the six orders was used twice to obtain a total of 12 orders. The 12 orders of the 12 CWIT versions were randomly allocated to the 12 participants.

#### 2.2.5 Statistical analyses

CWIT (s) outcomes were not normally distributed. For that reason, Wilcoxon signed-rank tests were used to compare CWIT performance between individual WBV conditions and the control condition.

Statistical significance was set at p<0.05. Bonferroni’s adjustment was applied to correct the statistical significance level for multiple comparisons. Moreover, effect sizes were calculated in the form of Pearson’s correlation coefficient r. Effect sizes were calculated as *r* = Z/√N using Wilcoxon signed-rank test statistics [21]. The N represents the total number of observations. Effect sizes were considered small when 0.10≤r<0.30, medium when 0.30≤r<0.50 and large when r≥0.50 [22]. Statistical analyses were performed with SPSS 17.0.

#### 2.2.6 Results

WBV condition 7 (30 Hz frequency and approximately 0.5 mm amplitude, see Table 1) was the only WBV condition that significantly (p = 0.004) improved CWIT (s) performance compared to the control condition (Table 2).

**Table 2:** Color-Word Interference Test (CWIT) performance after the control condition and after WBV conditions 1–10 (n = 12).

Condition	CWIT (s) (mean±SD)	% change compared to control	z-value	p-value^a^ (one-tailed)	Effect size (r)
Control	13.43±2.4	−	−	−	−
WBV 1	13.02±4.5	−3.1%	1.10	0.136	0.22
WBV 2	13.15±4.2	−2.1%	1.26	0.105	0.26
WBV 3	12.32±2.0	−8.3%	2.13	0.017	0.44
WBV 4	12.45±2.0	−7.3%	1.96	0.025	0.40
WBV 5	13.48±3.3	+0.3%	−0.24	0.593	−0.05
WBV 6	13.16±4.5	−2.0%	0.90	0.184	0.18
WBV 7	12.35±2.3	−8.0%	2.67	0.004^*^	0.54
WBV 8	12.66±2.4	−5.7%	1.96	0.025	0.40
WBV 9	12.52±2.5	−6.8%	2.04	0.021	0.41
WBV 10	12.73±2.9	−5.2%	1.65	0.050	0.33

Results of Wilcoxon signed-rank tests between WBV conditions and the control condition are also shown.^ a^ P-values <0.005 are significant after Bonferroni correction for multiple comparisons. *Indicates statistical significance.

#### 2.2.7 Conclusion

The passive WBV condition with 30 Hz frequency and approximately 0.5 mm amplitude (WBV condition 7 in Table 1) improved CWIT performance in healthy young adults. Therefore, this passive WBV condition was selected for further investigation in the main study.

### 2.3 The Main Study

#### 2.3.1 Participants

In total, 133 healthy young adults (112 females and 21 males; age: 20.5±2.2 years; mass: 65.5±11.1 kg; height: 1.73±0.8 m; amount of exercise: 3.3±3.3 hours/week) participated. All participants were students from the University of Groningen, the Netherlands. Participation was on a voluntary basis and not paid. However, participation was rewarded towards a research requirement for undergraduate students. None of the participants reported any history of neurological or psychiatric disease and none of the participants indicated to suffer from red-green color deficiency.

#### 2.3.2 Study design and procedures

Each participant underwent six times a passive WBV session with a frequency of 30 Hz and an amplitude of approximately 0.5 mm (WBV condition 7 in Table 1) and six times a control session while sitting on the WBV chair. The control session was without vibration. The order of the 12 sessions was counterbalanced across participants to avoid order effects. Figure 2 shows one of the two session orders used in the main study. Each session (passive WBV or control) lasted two minutes, immediately followed by assessments of executive functions and a rest period of three minutes while sitting on the WBV chair (without vibration). Following each rest period, participants underwent the next session (passive WBV or control).

During the whole experiment participants were sitting in an upright position against the back of the chair, with their arms positioned on the armrests and their feet on the wooden plate. Participants were asked to keep body movements to a minimum.

#### 2.3.3 Measurements

Participants performed the Color-Block Test (CBT) and the CWIT as conditions of the Stroop Color-Word Interference task [20]. The CBT is a measure of psychomotor speed and attention. In the CBT, 20 squares printed in one of four possible colors (blue, red, yellow or green) were presented on a card. The participant’s task was to name the colors of the squares as fast as possible. Time (in seconds) needed to complete the test was measured. The procedure of the CWIT was the same as in the pilot study, except that four colors (blue, red, yellow and green) were used instead of five colors. In addition, the Stroop Difference Score (SDS) was calculated as an additional measure of attention and inhibition by subtracting the time (in seconds) needed for completion of the CBT from the time (in seconds) needed for completion of the CWIT [20].

Participants also performed the Digit Span Backward task (DSBT). The DSBT is a subtest of the Wechsler Memory Scale-Revised and is a measure of executive functioning (working memory) [23]. In the DSBT a sequence of digits was read aloud to the participant with a speed of one digit per second. The task of the participant was to repeat the digits in the reversed order. Two sequences with the same number of digits were tested, one after the other. If at least one of the two sequences was recalled correctly in the reversed order, the level of difficulty was increased by adding one more digit to the length of the sequence. The first two sequences consisted of two digits. The test ended when a participant failed to recall both sequences of the same length in the reversed order. The number of correctly recalled sequences in reversed order was registered.

The participants practiced the CBT, the CWIT and the DSBT once before the start of the experiment in order to become familiar with the procedures of the tests. Immediately after each session (passive WBV or control), the CBT, the CWIT and the DSBT were performed (Figure 2). Following three passive WBV sessions and three control sessions, the CBT and the CWIT were performed before the DSBT (Figure 2). Following the other three passive WBV sessions and three control sessions, the DSBT was performed prior to the CBT and the CWIT (Figure 2). The CBT always preceded the CWIT. The order in which the Stroop tests and the DSBT were applied was counterbalanced across participants. In total 13 parallel versions (one version for practice and 12 versions for the assessments during the experiment) were designed of the CBT, the CWIT and the DSBT.

#### 2.3.4 Statistical analyses

Two different test sequences (first the DSBT and then the two conditions of the Stroop Color-Word Interference task or vice versa) were performed three times after two different experimental sessions (passive WBV or control). Therefore, per cognitive test a mean score was calculated across the three trials for each combination of experimental session and test sequence. All outcome measures were normally distributed. Cognitive performance was compared between the passive WBV session and the control session by using dependent t-tests.

A p-value smaller than 0.05 was considered to indicate statistical significance. Pearson’s correlation coefficient r was used as effect size measure and calculated as r = √(t^2^/(t^2^+df)) using dependent t-test statistics [21]. The effect sizes were interpreted as follows: small when 0.10≤r<0.30, medium when 0.30≤r<0.50 and large when r≥0.50 [22]. SPSS 17.0 was used to perform the statistical analyses.

#### 2.3.5 Results

*Cognitive performance immediately after passive WBV and control session:* CBT (s) results did not reveal differences (p = 0.16) between the passive WBV and control session (Table 3). However, performance on the CWIT (s) and the SDS (s) improved significantly (p = 0.009 and p = 0.034 respectively) after the passive WBV session compared to the control session (Table 3). The performance on the DSBT (number of correctly recalled sequences) did not differ (p = 0.66) between the passive WBV and control session (Table 3).

**Table 3:** Comparison of the performance on the Color-Block Test (CBT), Color-Word Interference Test (CWIT), Stroop Difference Score (SDS) and the Digit Span Backward task (DSBT) between the WBV and control session (n = 133).

	WBV session (mean±SD)	Control session (mean±SD)	t-value	p-value^a^	Effect size r
*Performance immediately after WBV and control session*					
CBT (s)	9.67±1.44	9.73±1.40	1.00	0.16	0.09
CWIT (s)	13.37±2.09	13.64±2.19	2.40	0.009*	0.20
SDS (s)	3.72±1.46	3.92±1.62	1.84	0.034*	0.16
DSBT (number)	6.24±1.92	6.29±1.76	−0.41	0.66	−0.04
*Performance after another cognitive test*					
CBT (s)	9.83±1.41	9.88±1.32	0.84	0.20	0.07
CWIT (s)	13.37±2.13	13.41±2.28	0.33	0.37	0.03
SDS (s)	3.54±1.44	3.53±1.58	−0.12	0.55	−0.01
DSBT (number)	6.22±1.58	6.13±1.93	0.85	0.20	0.07

a One-tailed p-value.

*p<0.05 indicates statistical significance.

*Cognitive performance when cognitive test was preceded by another cognitive test:* Performance on the CBT (s), CWIT (s) and SDS (s) did not differ (p≥0.20) between the passive WBV and control session (Table 3). Also performance on the DSBT (number of correctly recalled sequences) did not change (p = 0.20) by passive WBV compared to the control session (Table 3).

## Discussion

The general aim of this study was to investigate the acute effects of passive WBV on executive functions in healthy young adults. We hypothesized that passive WBV would improve executive functions in young adults, possibly as a result of increased neurotransmission in the prefrontal cortex by sensory stimulation. Our hypothesis was based on the neuroanatomical connections between mechanoreceptors and cognition-related brain regions [12], [13], and the positive effects of WBV on cognition and neurotransmitter systems in mice [15]–[17]. Indeed, the results of the pilot and main study showed that two minutes passive WBV with 30 Hz frequency and approximately 0.5 mm amplitude (WBV condition 7 in Table 1) improves CWIT performance in young adults, notwithstanding the already high level of cognitive functioning of the subjects. In addition, the main study demonstrated that two minutes passive WBV improved performance on the CWIT and the SDS only when the CBT and CWIT were performed immediately after passive WBV. Thus, two minutes passive WBV improved specific executive functions (attention and inhibition) measured with the CWIT and the SDS, while leaving another executive function (working memory) measured with the DSBT unchanged.

The improvement in CWIT performance by passive WBV may be explained by improved functioning of the prefrontal cortex and regions around the inferior frontal sulcus, because these brain regions are associated with CWIT performance [24]. Since afferent signals of cutaneous mechanoreceptors are transmitted to sensory brain areas [12] that are connected to prefrontal brain regions [13], passive WBV may acutely increase neurotransmission in the prefrontal cortex and in regions around the inferior frontal sulcus by sensory stimulation. Future research should investigate whether passive WBV increases neuronal activity in the mentioned brain regions, and whether sensory stimulation is indeed the mechanism underlying the positive acute effects of passive WBV on executive functions.

Only the passive WBV condition with 30 Hz frequency and approximately 0.5 mm amplitude improved executive functions (attention and inhibition). This may be explained by the specific sensitivity of Meissner corpuscles in the skin. Meissner corpuscles are especially sensitive to 30–40 Hz vibration [11]. Therefore, passive WBV with 30 Hz frequency and approximately 0.5 mm amplitude may induce a relatively strong stimulation of Meissner corpuscles in the skin, subsequently improving executive functioning. Other studies indicate that 30 Hz frequency is also optimal for stimulating oxygen uptake and muscle activity during active forms of WBV [5], [6].

Evidence for a short duration of the positive acute effects of passive WBV on executive functions is provided by the main study. Passive WBV did not improve performance on the CWIT and the SDS when the CBT and the CWIT were preceded by the DSBT (the duration of the DSBT was about two minutes). Additional evidence for a short duration of the positive acute effect of passive WBV on executive functions is provided by a comparison between the results of the main study and the results of the pilot study. In the main study the CWIT showed an improvement when it was performed only after the CBT, which was approximately 10 to 25 seconds after passive WBV. In the pilot study, however, the CWIT was performed directly after passive WBV. The positive effect on the CWIT was larger in the pilot study (effect size r = 0.54 versus r = 0.20). Therefore, the results of both studies indicate that the positive acute effects of passive WBV on executive functions are of short duration and already reduced in the time interval of approximately 10 to 25 seconds after passive WBV. This may explain the absence of improvements in DSBT performance, since the duration of the DSBT was approximately two minutes. Further research should investigate the effects of passive WBV on shorter tests of verbal working memory to find out whether verbal working memory is sensitive to the effects of passive WBV.

This short duration may question the functional relevance of our findings, but it should be noted that the optimal duration of a single passive WBV session for enhancing cognitive performance is unknown. In the present study the duration of the passive WBV sessions was very short (2 minutes). A longer acute exposure to passive WBV may increase the size of the positive effects on the CWIT and the SDS as well as the duration of the positive effects on executive functions. A longer passive WBV treatment may also improve performance on the DSBT. For these reasons it is recommended that future studies investigate the acute cognitive effects of prolonged periods of passive WBV (i.e. >2 minutes). This recommendation is supported by a recent pilot study with healthy adults in which we used a duration of 4 minutes of passive WBV in a chronic treatment protocol (5 weeks, 3 days per week, n = 5). Our preliminary result revealed even stronger improvements in Stroop test performance (maximally 14% improvement) than the acute effects reported here. Moreover, the effects were still significantly present after one week without WBV treatment, illustrating the potential of passive WBV to improve cognition.

The results of this study indicate that passive WBV may be a clinically relevant therapy. We found that passive WBV enhances CWIT performance in healthy young adults with a high level of education and an age at which CWIT performance is highest across the lifespan [25]. This suggests that passive WBV may also have treatment effects in older adults and other populations with impaired attention and inhibition (such as people with attention deficit hyperactivity disorder). The forementioned pilot study on a chronic, 4 minutes passive WBV treatment in healthy adults corroborates this suggestion. Furthermore, passive WBV is easy to apply and not expensive. Therefore, passive WBV may be a relevant therapy for cognitively impaired populations unable to perform active forms of exercise, such as immobile older adults. Future studies should investigate the cognitive effects of passive WBV in older persons and clinical populations.

A limitation of this study may be the lack of an appropriate control condition. In the control sessions participants were sitting on the switched-off WBV chair, without the sound of the switched-on WBV platform. This suggests that the sound produced by the WBV platform may have contributed to the observed positive effects on cognition. However, the sound of the WBV condition that enhanced cognitive performance was very similar to the sound of other WBV conditions that did not improve cognitive performance in the pilot study. Therefore, results of the pilot study indicate that it is primarily the vibration that enhances cognitive performance and not the sound of the WBV platform. A strong point of the main study was the lack of an untreated control condition, since participants underwent passive WBV a couple of minutes before the control conditions. This is a conservative approach and strengthens the differences in cognitive performance that we found between the passive WBV and control condition. Other strong points of the main study were the use of an optimal vibration frequency and amplitude for improving executive functions based on the results of the pilot study, and the large sample size (n = 133).

In conclusion, this study demonstrated that passive WBV has positive effects on cognition in humans. Results showed that a short session of passive WBV with 30 Hz frequency and approximately 0.5 mm amplitude has a positive short-term effect on executive functions (attention and inhibition) in healthy young adults with a high level of cognitive functioning. Additional studies are in progress in young adults, (immobile) older adults and people with attention deficit hyperactivity disorder.
